# Identification of C-PLAN index as a novel prognostic predictor for advanced lung cancer patients receiving immune checkpoint inhibitors

**DOI:** 10.3389/fonc.2024.1339729

**Published:** 2024-02-08

**Authors:** Jiaxin Wang, Huaijuan Guo, Jingjing Yang, Jingxian Mao, Ying Wang, Xuebing Yan, Hong Guo

**Affiliations:** ^1^ Department of Oncology, The Affiliated Hospital of Yangzhou University, Yangzhou University, Yangzhou, China; ^2^ Department of Thoracic Surgery, The Affiliated Hospital of Yangzhou University, Yangzhou University, Yangzhou, China

**Keywords:** immune checkpoint inhibitor, lung cancer, C-PLAN, prognosis, biomarker

## Abstract

**Objective:**

Increasing studies have highlighted the potential utility of non-invasive prognostic biomarkers in advanced lung cancer patients receiving immune checkpoint inhibitor (ICI) based anti-cancer therapies. Here, a novel prognostic predictor named as C-PLAN integrating C-reactive protein (CRP), Performance status (PS), Lactate dehydrogenase (LDH), Albumin (ALB), and derived Neutrophil-to-lymphocyte ratio (dNLR) was identified and validated in a single-center retrospective cohort.

**Methods:**

The clinical data of 192 ICI-treated lung cancer patients was retrospectively analyzed. The pretreatment levels of CRP, PS, LDH, ALB and dNLR were scored respectively and then their scores were added up to form C-PLAN index. The correlation of C-PLAN index with the progression-free survival (PFS) or overall survival (OS) was analyzed by a Kaplan–Meier model. The multivariate analysis was used to identify whether C-PLAN index was an independent prognostic predictor.

**Results:**

A total of 88 and 104 patients were included in the low and high C-PLAN index group respectively. High C-PLAN index was significantly correlated with worse PFS and OS in ICI-treated lung cancer patients (both p<0.001). The multivariate analysis revealed high C-PLAN index was an independent unfavorable factor affecting PFS (hazard ratio (HR)=1.821; 95%confidence interval (CI)=1.291-2.568) and OS (HR=2.058, 95%CI=1.431-2.959). The high C-PLAN index group had a significantly lower disease control rate than the low C-PLAN index group (p=0.024), while no significant difference was found for objective response rate (p=0.172). The subgroup analysis based on clinical features (pathological type, therapy strategy, TNM stage and age) confirmed the prognostic value of C-PLAN index, except for patients receiving ICI monotherapy or with age ranging from 18 to 65 years old. Finally, a nomogram was constructed based on C-PLAN index, age, gender, TNM stage and smoking status, which could predict well the 1-, 2- and 3-year survival of ICI-treated lung cancer patients.

**Conclusion:**

The C-PLAN index has great potential to be utilized as a non-invasive, inexpensive and reliable prognostic predictor for advanced lung cancer patients receiving ICI-based anti-cancer therapies.

## 1 Introduction

Lung cancer is a commonly diagnosed human malignancy worldwide, ranking the first in cancer-related mortality among all the cancer types (1). The traditional therapy paradigm for lung cancer includes surgery, chemoradiotherapy and targeted therapy, while its five-year relative survival is only approximately 23% (2). The introduction of immune checkpoint inhibitor (ICI) based immunotherapy holds promise for advanced cancer patients, and its durable efficacy with controllable adverse events is observed in accumulating clinical trials (3). The pharmacological mechanism of ICIs is inhibition of cellular receptors including programmed cell death protein 1 (PD-1), programmed death ligand-1 (PD-L1) and cytotoxic T lymphocyte-associated antigen 4 (CTLA-4), known as representative drugs such as Pembrolizumab, Durvalumab and Ipilimumab respectively (4). Despite encouraging achievements, some evidences have suggested only less than half of patients benefit from ICI therapy and few patients even experience tumor hyperprogression at the initial stage (5). Currently, various factors are found to affect the clinical efficacy of ICI drugs such as PD-L1 expression, tumor mutation burden (TMB), microbiome and immune infiltration (6). Previously, our team has proved the detrimental effect of several concomitant drugs on ICI efficacy (7–9). A further investigation into the factors affecting ICI efficacy will contribute to developing novel predictive strategies for patient outcome, leading to more tailored therapy decision.

Recently, emerging studies have suggested some clinical parameters could be utilized as non-invasive and low-cost biomarkers for predicting ICI-treated lung cancer patients. For instance, the neutrophil-to-lymphocyte ratio (NLR) has been identified as a prognostic predictor for non-small-cell lung cancer (NSCLC) patients receiving combined immunotherapy and chemotherapy, regardless of PD-L1 expression (10). The early treatment levels of C-reactive protein (CRP) and lactate dehydrogenase (LDH), instead of their pretreatment baseline levels, are found to significantly associate with one-year OS of ICI-treated NSCLC patients (11). The modified Glasgow prognostic score (mGPS), determined by CRP and albumin (ALB) level, could predict the OS and progression-free survival (PFS) of NSCLC patients receiving ICI consolidation after chemoradiotherapy (12). The systemic immune-inflammation index (SII), combining platelet count and NLR, is an unfavorable factor affecting the PFS of NSCLC patients receiving PD-1 inhibitors (13). Our team has previously proved prognostic nutritional index (PNI) as a reliable prognostic indicator for both ICI-treated NSCLC and small cell lung cancer (SCLC) patients (14). However, these prognostic indexes are mainly based on laboratory tests, ignoring individual status such as performance status (PS) and smoking status, which may be also crucial for ICI efficacy (15, 16). Therefore, one or two parameters are far from sufficient to precisely stratify the outcome of ICI-treated NSCLC patients and novel predictive methods are urgently needed.

A recent multicenter retrospective study has developed a comprehensive scoring system that integrates CRP, LDH, ALB, dNLR and PS (named as C-PLAN index) to predict the prognosis of NSCLC patients receiving immunotherapy combined with other anti-cancer therapies (17). In this study, an independent cohort was utilized to further investigate the prognostic value of C-PLAN index in ICI-treated lung cancer patients. The study will not only contribute to validating a novel prognostic index for patient management, but also highlight the potential utility of integrating multiple clinical parameters in outcome prediction.

## 2 Methods and materials

### 2.1 Patient recruitment

**Figure 1:**
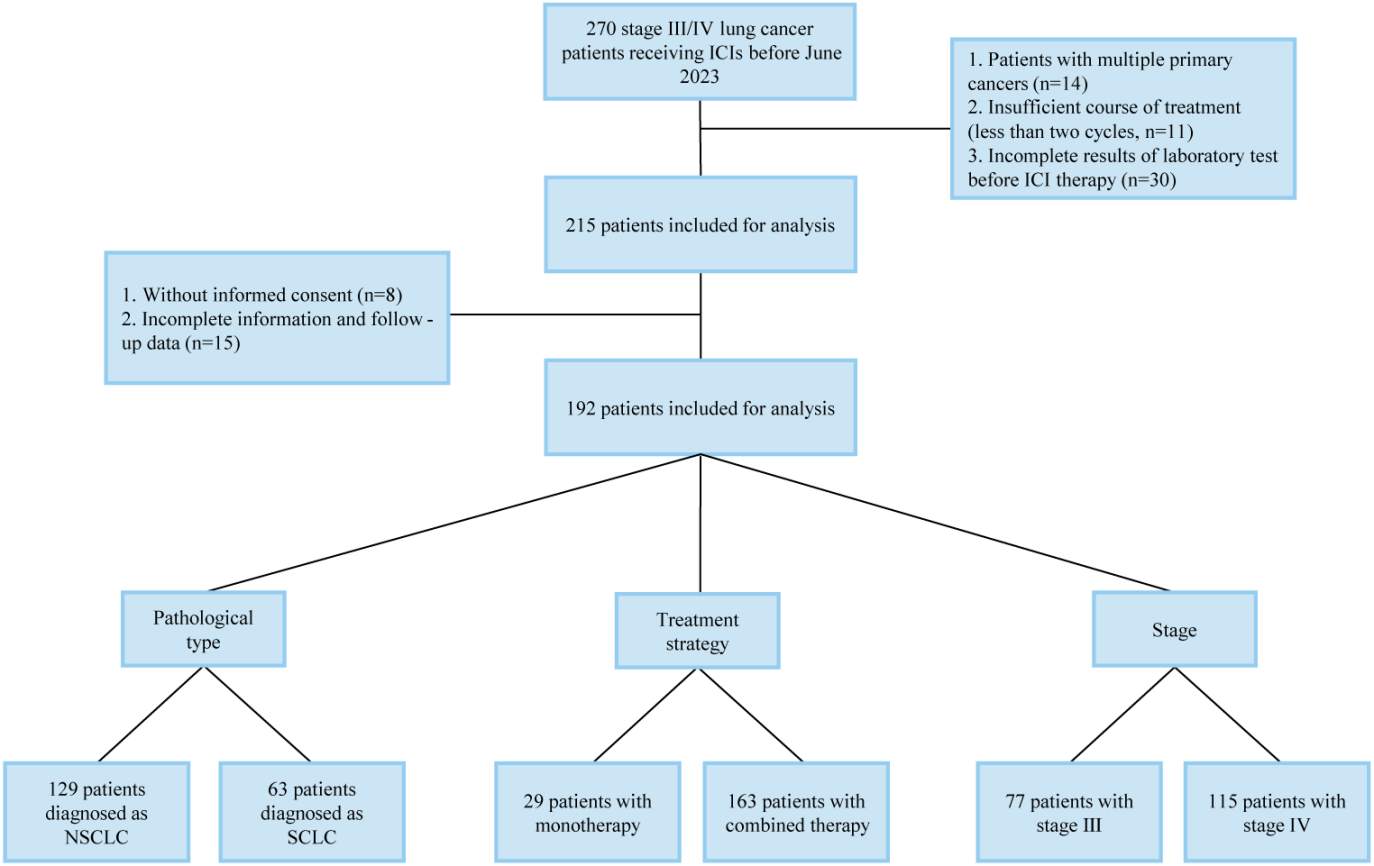
Flowchart of the patient recruitment in the retrospective cohort.

The flow chart of patient recruitment is shown in **Figure 1**. The inclusion criteria are as follows: 1) age over 18 years; 2) diagnosed as stage III/IV lung cancer; 3) receiving ICIs alone or combined with other anti-cancer therapies. The exclusion criteria are as follows: 1) multiple primary tumors; 2) incomplete laboratory tests before ICI therapy; 3) insufficient ICI therapy (less than two cycles); 4) incomplete therapy or follow-up records; 5) unavailable informed consents. All the enrolled patients received anti-cancer therapies at the department of oncology, Affiliated Hospital of Yangzhou University from June 2019 to June 2023. Tumor stage was classified according to TNM staging system (AJCC 8^th^ Edition). The PS was scored according to the Eastern Cooperative Oncology Group (ECOG) criterion. This study was approved by the ethics committee of the hospital (No. 2022-YKL11-class 05) and informed consents were obtained from patients for using their information in medical studies.

### 2.2 Oncological evaluation and follow-up

For oncological evaluation, blood marker detection and radiological examination were performed every cycle and every two or three cycles respectively. The responses of anti-cancer therapies were determined using complete response (CR), partial response (PR), progressive disease (PD), and stable disease (SD), all of which were evaluated according to Response Evaluation Criteria in Solid Tumors (RECIST) 1.1. Objective response rate (ORR) was defined as the proportion of the CR+PR, while disease control rate (DCR) was defined as the proportion of CR+PR+SD. The clinical outcome of patients was determined using OS and PFS. The OS was defined as the time interval between the first ICI therapy initiation and death caused by any reasons, while the PFS was defined as the time interval between the first ICI therapy initiation and disease progression.

### 2.3 Definition of C-PLAN index

The C-PLAN index was scored as described previously (17). In brief, the C-PLAN index was calculated as the sum score of CRP, LDH, ALB, dNLR and PS, which were respectively scored as follows: score 0 (CRP<1.0mg/dL or LDH<223U/L or ALB≥3.5g/dL or derived NLR<3.0 or PS ≤ 1); score 1(CRP≥1.0mg/dL or LDH≥223U/L or ALB<3.5g/dL or derived NLR≥3.0 or PS>1). The patients with the C-PLAN index ≤ 1 were allocated into the low index group, while the rest were allocated into the high index group. Since some pathological and physiological conditions (such as infections) are known to significantly affect the levels of these markers, therefore the clinical conditions of patients were sufficiently considered before immunotherapy initiation and pre-treatment marker detection.

### 2.4 Statistical analysis

The statistical analysis was performed using SPSS Statistics (version 25.0) or GraphPad Prism (version 8.0) or R package (version 4.3.0). The correlations of clinical parameters with the C-PLAN index were determined using chi-square test. The OS and PFS curves were plotted using a Kaplan–Meier model, and analyzed using the log-rank test. The univariate and multivariate analysis were used to identify significant independent prognostic factors. A p value less than 0.05 was considered to be statistically significant.

## 3 Results

### 3.1 General description of patient characteristics

According to the inclusion and exclusion criteria, a total of 192 patients were finally included in the study and their characteristics were shown in **Table 1**. The median age of the cohort was 69 years old, ranging from 51 to 86 years old. 129 (67.2%) and 63 (32.8%) patients were pathologically confirmed as NSCLC and SCLC respectively. 37 patients (19.3%) received surgical resection before ICI therapy. 99 patients (51.6%) had smoking history. 77 (40.1%) and 115 (59.9%) patients were diagnosed as stage III and stage IV disease respectively. 163 patients (84.9%) received combined anti-cancer therapies, while the rest received ICI monotherapy. The ICI drugs were used as follows: sintilimab (n=68), tirelizumab (n=38), camrelizumab (n=25), durvalumab (n=18), serplulimab (n=14), pembrolizumab (n=11), atezolizumab (n=6), nivolumab (n=4), penpulimab (n=3), toripalimab (n=2), envafolimab (n=2) and adebrelimab (n=1). The chemotherapy drugs were used as follows: platinum (n=114), nab-paclitaxel/paclitaxel (n=46), etoposide (n=44), pemetrexed (n=23), docetaxel (n=12), irinotecan (n=4) and adriamycin (n=1). Targeted drugs were used in 28 patients, with apatinib for 1 patient, bevacizumab for 4 patients and anlotinib for 23 patients. 10 patients received radiotherapy.

**Table 1:** Baseline characteristics for the included patients.

Factors	Total	Low C-PLAN (n=88)	High C-PLAN (n=104)	X^2^	p-value
Gender					
Female	19(9.9)	9(10.2)	10(9.6)	0.020	0.887
Male	173(90.1)	79(89.8)	94(90.4)		
Age					
≤65 years old	56(29.2)	31(35.2)	25(24.0)	2.888	0.089
>65 years old	136(70.8)	57(64.8)	79(76.0)		
Histological type					
SCLC	63(32.8)	27(30.7)	36(34.6)	0.335	0.563
NSCLC	129(67.2)	61(69.3)	68(65.4)		
Surgery history					
No	155(80.7)	68(77.3)	87(83.7)	1.248	0.264
Yes	37(19.3)	20(22.7)	17(16.3)		
Staging					
III	77(40.1)	40(45.5)	37(35.6)	1.936	0.164
IV	115(59.9)	48(54.5)	67(64.4)		
PS					
0-1	165(85.9)	84(95.5)	81(77.9)	12.176	<0.001
2-3	27(14.1)	4(4.5)	23(22.1)		
Smoking					
Never	93(48.4)	41(46.6)	52(50.0)	0.222	0.638
Current/former	99(51.6)	47(53.4)	52(50.0)		
Treatment					
Monotherapy	29(15.1)	10(11.4)	19(18.3)	1.773	0.183
Combination	163(84.9)	78(88.6)	85(81.7)		

SCLC, small cell lung cancer; NSCLC, non-small cell lung cancer; PS, performance status.

The correlation analysis demonstrated the C-PLAN index was significantly correlated with PS (p<0.001), while no significant correlation was observed between C-PLAN index and other clinical characteristics including gender (p=0.887), age (p=0.089), histological type (p=0.563), surgery history (p=0.264), TNM stage (p=0.164), smoking history (p=0.638) and therapy strategy (p=0.183).

### 3.2 Prognostic significance of the C-PLAN index in the entire cohort

**Figure 2:**
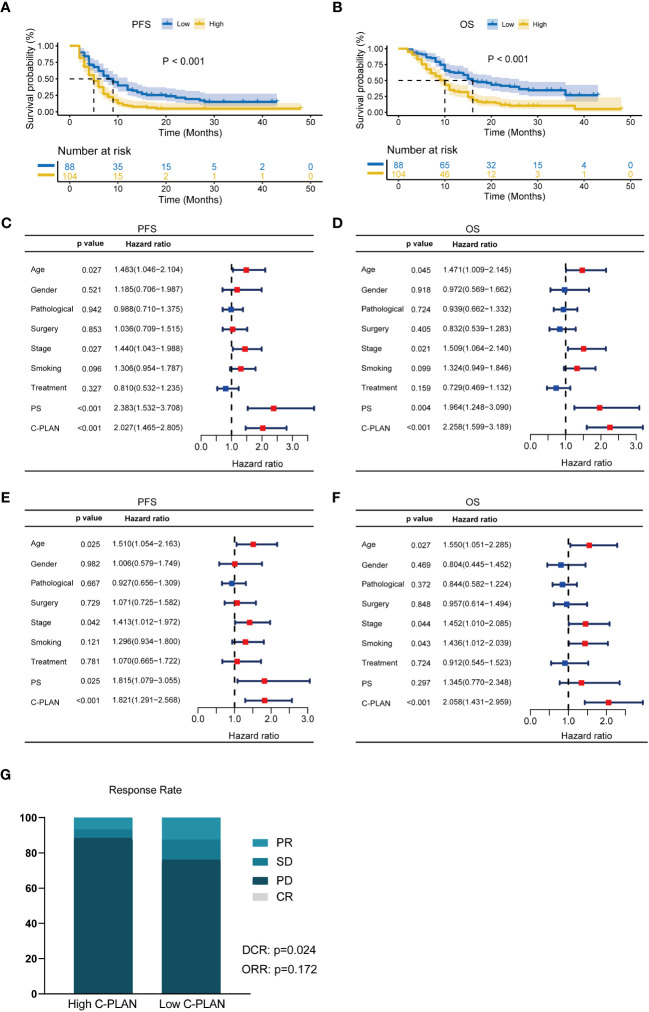
Prognostic significance of C-PLAN index in advanced lung cancer patients receiving immune checkpoint inhibitors (ICIs). **(A, B)** Kaplan-Meier curves for the association of C-PLAN index with progression-free survival (PFS) **(A)** and overall survival (OS) **(B)** in advanced lung cancer patients receiving ICIs. **(C, D)** Univariate analysis for identifying the prognosis factors significantly correlated with the PFS **(C)** and OS **(D)** in advanced lung cancer patients receiving ICIs. **(E, F)** Multivariate analysis for identifying the significantly independent prognosis factors for PFS **(E)** and OS **(F)** in advanced lung cancer patients receiving ICIs. **(G)** Correlations of C-PLAN index with therapy response in advanced lung cancer patients receiving ICIs.

The Kaplan–Meier curves of the PFS and OS for the entire cohort were shown in **Figures 2A, B**. The patients with high C-PLAN index had a significantly worse PFS and OS than those with low C-PLAN index (PFS: p<0.001; OS: p<0.001). In the univariate analysis (**Figures 2C, D**), age, stage, PS and C-PLAN were significant factors affecting PFS and OS. The multivariate analysis identified high C-PLAN index was an independent negative predictor for PFS and OS (**Figures 2E, F**).

In terms of therapy response (**Figure 2G**), 11 (12.5%) and 77(87.5%) patients were diagnosed as PR and SD/PD respectively in the low C-PLAN index group, as compared with 7 (6.7%) and 97 (93.3%) patients in the high C-PLAN index group. The further analysis revealed the patients with low C-PLAN index had a significantly higher DCR instead of ORR than those with high C-PLAN index (DCR: p=0.024; ORR: p=0.172).

### 3.3 Prognostic significance of the C-PLAN index in the subgroup analysis

**Figure 3:**
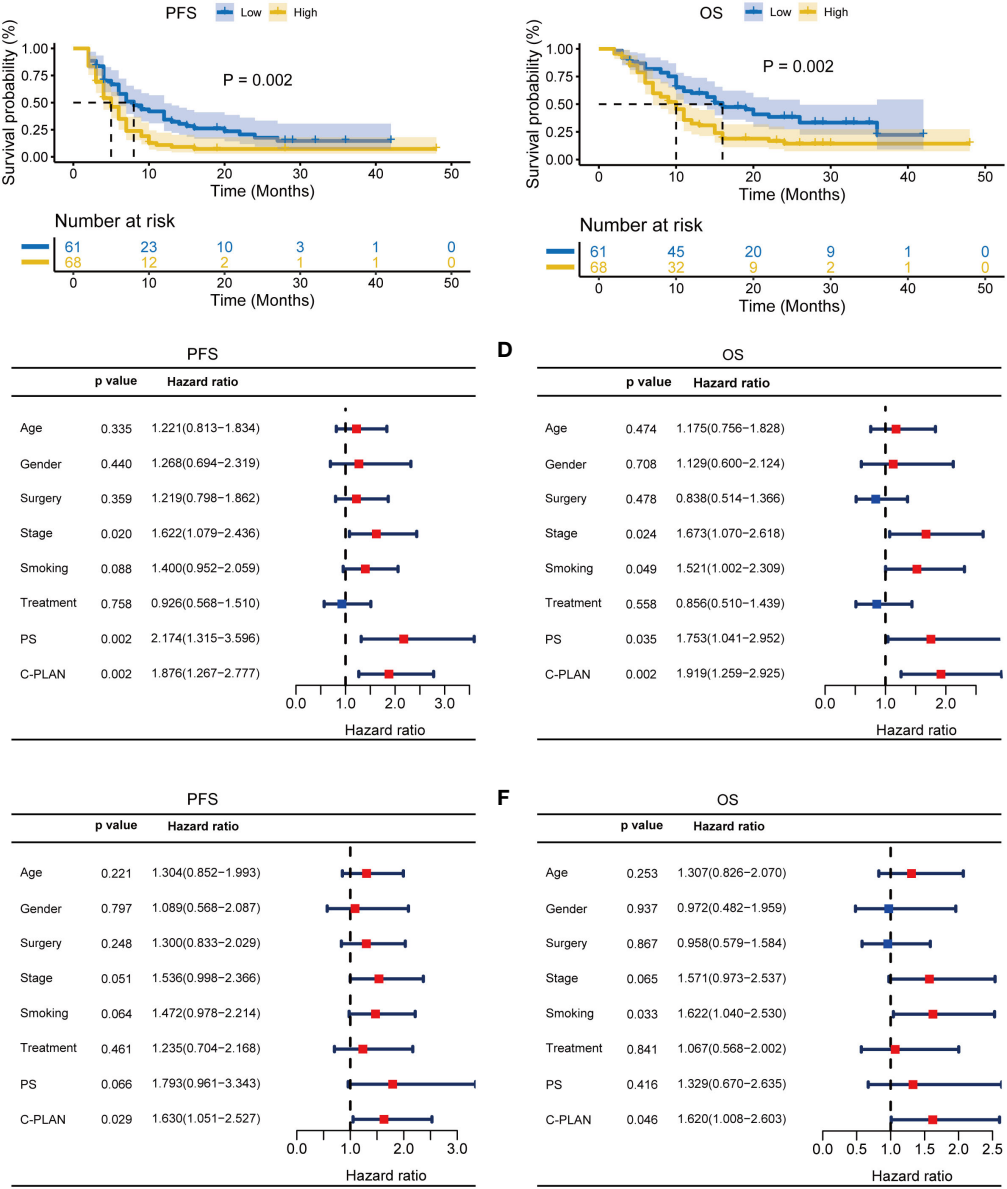
Prognostic significance of C-PLAN index in non-small cell lung cancer (NSCLC) patients receiving immune checkpoint inhibitors (ICIs). **(A, B)** Kaplan-Meier curves for the association of C-PLAN index with progression-free survival (PFS) **(A)** and overall survival (OS) **(B)** in NSCLC patients receiving ICIs. **(C, D)** Univariate analysis for identifying the prognosis factors significantly correlated with the PFS **(C)** and OS **(D)** in NSCLC patients receiving ICIs. **(E, F)** Multivariate analysis for identifying the significantly independent prognosis factors for PFS **(E)** and OS **(F)** in NSCLC patients receiving ICIs.

For further clarifying the prognostic significance of the C-PLAN index in the ICI-treated lung cancer patients, the subgroup analysis was performed based on NSCLC and SCLC. As shown in **Figures 3A, B**, high C-PLAN index was significantly correlated with worse PFS and OS of ICI-treated NSCLC patients (PFS: p=0.002; OS: p=0.002). The univariate analysis demonstrated stage, PS and C-PLAN were significantly correlated with PFS (**Figure 3C**), while these factors and smoking history were significantly correlated with OS (**Figure 3D**). The following multivariate analysis identified high C-PLAN index was an independent adverse predictor for PFS and OS (**Figures 3E, F**). Regarding SCLC patients, the negative association of C-PLAN index with patient outcomes was also significant (PFS: p=0.002, **Supplementary Figure S1A**; OS: p<0.001, **Supplementary Figure S1B**). The univariate analysis indicated age, treatment strategy, PS and C-PLAN were significant factors affecting PFS and OS (**Supplementary Figures S1C, D**). The multivariate analysis confirmed both PS and C-PLAN index were independent predictors for PFS and OS (**Supplementary Figures S1E, F**).

**Figure 4:**
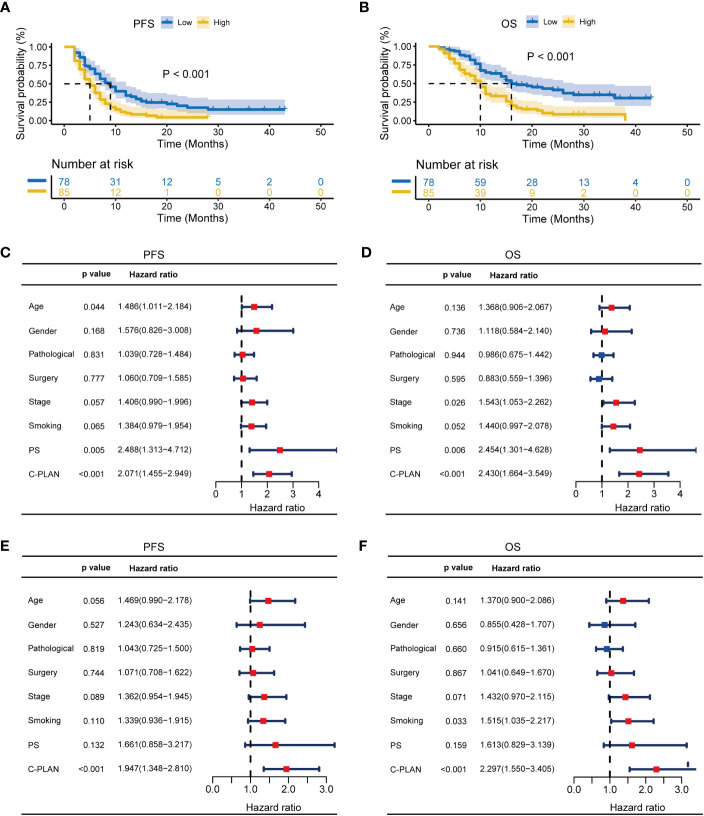
Prognostic significance of C-PLAN index in advanced lung cancer patients receiving combined therapies. **(A, B)** Kaplan-Meier curves for the association of C-PLAN index with progression-free survival (PFS) **(A)** and overall survival (OS) **(B)** in advanced lung cancer patients receiving combined therapies. **(C, D)** Univariate analysis for identifying the prognosis factors significantly correlated with the PFS **(C)** and OS **(D)** in advanced lung cancer patients receiving combined therapies. **(E, F)** Multivariate analysis for identifying the significantly independent prognosis factors for PFS **(E)** and OS **(F)** in advanced lung cancer patients receiving combined therapies.

The subgroups were also classified according to combined therapy or monotherapy. For patients receiving combined therapy, high C-PLAN index group had significantly worse PFS and OS than low C-PLAN index group (PFS: p<0.001, **Figure 4A**; OS: p<0.001, **Figure 4B**). The C-PLAN index together with age and PS were identified as significant factors affecting PFS (**Figure 4C**), while the C-PLAN together with stage and PS were identified as significant factors affecting OS (**Figure 4D**). The C-PLAN index was also found to be an independent prognostic factor for patients receiving combined therapy (**Figures 4E, F**). For patients receiving monotherapy, neither PFS nor OS significantly differed between high and low C-PLAN index group (PFS: p=0.248, **Supplementary Figure S2A**; OS: p=0.461, **Supplementary Figure S2B**). Consistently, the C-PLAN index was not a significant factor affecting PFS and OS in the univariate analysis (**Supplementary Figures S2C, D**).

Finally, the subgroups were classified according to stage III or stage IV. For stage III patients, the negative correlation between C-PLAN index and patient outcome remained statistically significant (PFS: p=0.032, **Supplementary Figure S3A**; OS: p<0.001, **Supplementary Figure S3B**). This correlation was also confirmed by the univariate analysis (**Supplementary Figures S3C, D**) and it was further identified as an independent negative predictor for OS (**Supplementary Figure S3F**) rather than PFS (**Figure S3E**). For stage IV patients, similar results were also found in the Kaplan–Meier survival analysis (PFS: p<0.001, **Supplementary Figure S4A**; OS: p=0.001, **Supplementary Figure S4B**). The univariate analysis demonstrated age, PS and the C-PLAN index were significantly correlated with PFS (**Supplementary Figure S4C**), while the C-PLAN index was the only significant factor affecting OS (**Supplementary Figure S4D**). The multivariate analysis revealed the C-PLAN index and age were independent predictors for PFS (**Supplementary Figure S4E**), while only the C-PLAN index was for OS (**Supplementary Figure S4F**). With regard for the subgroups stratified by age, the prognostic value of the C-PLAN index was successfully confirmed in patients with age over 65 years old (**Supplementary Figures S5A–F**). However, for patients with age ranging from 18 to 65 years, no significant difference was observed in PFS or OS between the two groups (**Supplementary Figures S6A, B**). The following univariate analysis also suggested C-PLAN index was unrelated with the PFS or OS of the patients.

### 3.4 Construction and validation of a prognostic nomogram based on the C-PLAN index

**Figure 5:**
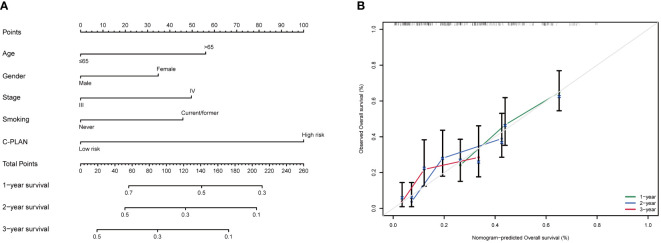
Construction of a prognostic nomogram based on C-PLAN index and clinical parameters. **(A)** A nomogram integrating C-PLAN index and clinical parameters predicts the 1-, 2-, and 3-year OS of advanced lung cancer patients receiving immune checkpoint inhibitors (ICIs). **(B)** Calibration curves validate the predictive accuracy of the nomogram in the 1-, 2-, and 3-year OS of advanced lung cancer patients receiving ICIs.

For further utilization of C-PLAN in prognostic prediction, a nomogram was established based on C-PLAN and other clinical parameters, including age, gender, stage and smoking history, which predicts the 1-, 2-, and 3-year OS of ICI-treated lung cancer patients (**Figure 5A**). The calibration curve model was successfully established to validate the reasonable concordance between the predicted and actual survival (**Figure 5B**).

## 4 Discussion

Although a great paradigm shift has been made by ICIs in the anti-cancer therapeutic scenario, their real benefit remains controversial and limited predictive biomarkers can be used for therapy decisions (18). PD-L1 is currently identified as a powerful molecular biomarker to guide ICI use, however, its accuracy may vary greatly with sample type and immunohistochemistry evaluation (19, 20). The advancing sequencing technique has provided TMB as a promising predictive biomarker for ICI therapy, but methodological differences and high cost limit its actual utilization (21). Accumulating evidences have closely linked gut microbiome with immunotherapy efficacy in lung cancer, while identifying prognosis-related bacteria and developing unified standards for detection methods are still challenging (22). Moreover, emerging studies have highlighted the potential utility of blood inflammatory or nutritional markers as prognostic markers, but their actual predictive performance may be far from satisfactory in metastatic NSCLC patients receiving first-line ICI therapy (23). Therefore, development and validation of novel, cost-effective and non-invasive biomarkers for ICI-treated lung cancer patients is an urgent task for clinicians. A recent multicenter study for the first time has integrated PS status, inflammatory and nutritional markers into a novel biomarker named as C-PLAN index, which was found to effectively stratify the outcome of NSCLC patients receiving immunochemotherapy (17). In this study, a single-center retrospective study including both NSCLC and SCLC patients was performed to further validate whether C-PLAN could act as a reliable prognostic biomarker for ICI-treated lung cancer patients.

For our entire cohort, patients with high C-PLAN index had a significantly worse PFS and OS than those with low C-PLAN index. The high C-PLAN index was associated with lower DCR as compared with low C-PLAN index. More importantly, the negative correlation of C-PLAN index with clinical outcome remained statistically significant in the multivariate analysis excluding other known risk factors such as tumor stage. These findings collectively confirmed C-PLAN index as an actionable prognostic predictor for ICI-treated lung cancer patients. The C-PLAN index includes following parameters: CRP, LDH, ALB, dNLR and PS score, all of which have been proved to associate with the clinical efficacy of ICI-based therapy. A meta-analysis enrolling 4698 patients has suggested higher pretreatment CRP level is correlated with worse OS and PFS in NSCLC patients receiving ICI therapy (24). The LDH level is found to significantly affect OS and PFS of NSCLC patients receiving nivolumab, although it fails to be proved as an independent prognostic factor in the multivariate analysis (25). In a real-world study, albumin <3.5 g/dL is identified as a crucial unfavorable prognostic factor in stage IV NSCLC patients receiving the first-line immunotherapy (26). High pre-treatment NLR level predicts poor outcome in advanced NSCLC patients receiving first-line pembrolizumab therapy (27). In KRASG12C-Mutant NSCLC patients receiving chemo-immunotherapy, poor PS is an independent unfavorable factor affecting OS and PFS (28). The PS ≥2 is also correlated with poor outcome in 70+ year-old lung cancer patients receiving pembrolizumab alone or with chemotherapy (29). Considering their potential prognostic value, the C-PLAN index combined and optimized these different factors to help clinicians effectively select patients who may benefit from ICI-based therapy.

For further clarifying the prognostic significance of C-PLAN index, the subgroup analysis was performed based on several major clinical features including pathological type, therapy strategy, TNM stage and age. The result identified C-PLAN index as an independent adverse prognostic factor for OS and PFS, regardless of pathological type and TNM stage, strongly supporting its clinical utilization. It is worth mentioning that the C-PLAN index fails to stratify the clinical outcome of patients receiving ICIs alone and patients with age ranging from 18 to 65 years old, which may be partly attributed to the limited sample size. To our knowledge, some recent studies have suggested potential novel prognostic biomarkers for these patients. For instance, RNA-binding motif protein 17 (RBM17) expression in tumor samples could be used to predict the clinical efficacy of ICI monotherapy in NSCLC patients with a low PD-L1 expression (30). Pre-treatment plasma levels of cachexia related immune mediators (such as osteopontin and pentraxin‐3) are found to associate with outcome of advanced or recurrent NSCLC patients receiving PD‐1/PD‐L1 inhibitor monotherapy (31). Increased non-classical monocytes are correlated with favorable outcome in NSCLC patients receiving anti-PD-1 antibody monotherapy, while the opposite was observed for PD-L1-expressing classical monocytes (32). In future, these biomarkers need to be validated in more retrospective cohorts and may be considered to combine with the C-PLAN index in outcome prediction.

Some recent mechanism investigations can be used to partly explain the correlation between C-PLAN index and ICI efficacy. Elevated pretreatment CRP may contribute to the production of adenosine 2a receptor that subsequently suppresses antitumor immune cell functions and upregulates immunosuppressive genes (33). High LDH level usually indicates enhanced glycolytic activity in tumors, resulting in glucose deprivation and tumor acidity to hamper antitumor immunity (34). In terms of drug pharmacokinetics, ICI drugs are mainly developed from IgG antibodies, and albumin participates in the catabolism and recycling of IgG antibodies through its binding with neonatal Fc receptor (35). The transcriptomic analysis reveals low baseline NLR level is correlated with increased expression of CD3, SH2D1A, ZAP70 and CD45RA, all of which are involved in immune activation (36). With regard to PS, lung cancer patients with PS > 2 are more likely to have serious comorbidities such as chronic obstructive pulmonary disease (COPD) that needs long-term corticosteroid use. Dexamethasone as a representative corticosteroid drug is found to inhibit the functions of activated T lymphocytes through upregulating PD-1 expression, finally diminishing ICI efficacy (37). Considering their potential regulatory role in tumor immunity, whether some interventions such as nutrition support and physical exercise could benefit ICI efficacy through improving these markers is worth further investigations.

Several limitations in our present study should be noted. Firstly, the sample size is relatively small and multicenter validations are essential in future. Secondly, due to the retrospective nature of the study, various factors such as patient selection, ICI drug type and treatment strategy may lead to heterogeneity. For instance, the sample size of the female patients in our study was limited (n=19), which may serve as a confounding variable. Therefore, more attention should be paid to the actual prognostic value of C-PLAN in subgroups such as female patients or patients receiving ICIs alone. Thirdly, whether C-PLAN index is correlated with the adverse events of combined therapy or ICI monotherapy remains unclear. Finally, the study mainly focused on the prognostic value of pretreatment C-PLAN index and whether its dynamic evaluation during therapy has any benefit for patient management also needs to be clarified.

In summary, our study identified C-PLAN index as a novel prognostic predictor for advanced lung cancer patients receiving ICI-based anti-cancer therapies. In future, more retrospective validations are essential and the clinical utility of C-PLAN index in ICI-treated patients with other cancers needs to be investigated.

## Data availability statement

The original contributions presented in the study are included in the article/**Supplementary Material**. Further inquiries can be directed to the corresponding authors.

## Ethics statement

The studies involving humans were approved by Affiliated Hospital of Yangzhou University. The studies were conducted in accordance with the local legislation and institutional requirements. The participants provided their written informed consent to participate in this study.

## Author contributions

JW: Conceptualization, Writing – original draft. HJG: Data curation, Formal analysis, Writing – original draft. JY: Resources, Software, Writing – review & editing. JM: Validation, Visualization, Writing – review & editing. YW: Data curation, Writing – review & editing. XY: Funding acquisition, Writing – review & editing. HG: Conceptualization, Writing – review & editing.

## Supplementary material

Supplementary Figure 1 Prognostic significance of C-PLAN index in small cell lung cancer (SCLC) patients receiving immune checkpoint inhibitors (ICIs). **(A, B)** Kaplan-Meier curves for the association of C-PLAN index with progression-free survival (PFS) **(A)** and overall survival (OS) **(B)** in SCLC patients receiving ICIs. **(C, D)** Univariate analysis for identifying the prognosis factors significantly correlated with the PFS **(C)** and OS **(D)** in SCLC patients receiving ICIs. **(E, F)** Multivariate analysis for identifying the significantly independent prognosis factors for PFS **(E)** and OS **(F)** in SCLC patients receiving ICIs.

Supplementary Figure 2 Prognostic significance of C-PLAN index in advanced lung cancer patients receiving immune checkpoint inhibitor (ICI) monotherapy. **(A, B)** Kaplan-Meier curves for the association of C-PLAN index with progression-free survival (PFS) **(A)** and overall survival (OS) **(B)** in advanced lung cancer patients receiving ICI monotherapy. **(C, D)** Univariate analysis for identifying the prognosis factors significantly correlated with the PFS **(C)** and OS **(D)** in advanced lung cancer patients receiving ICI monotherapy.

Supplementary Figure 3 Prognostic significance of C-PLAN index in stage III lung cancer patients receiving immune checkpoint inhibitors (ICIs). **(A, B)** Kaplan-Meier curves for the association of C-PLAN index with progression-free survival (PFS) **(A)** and overall survival (OS) **(B)** in stage III lung cancer patients receiving ICIs. **(C, D)** Univariate analysis for identifying the prognosis factors significantly correlated with the PFS **(C)** and OS **(D)** in stage III lung cancer patients receiving ICIs. **(E, F)** Multivariate analysis for identifying the significantly independent prognosis factors for PFS **(E)** and OS **(F)** in stage III lung cancer patients receiving ICIs.

Supplementary Figure 4 Prognostic significance of C-PLAN index in stage IV lung cancer patients receiving immune checkpoint inhibitors (ICIs). **(A, B)** Kaplan-Meier curves for the association of C-PLAN index with progression-free survival (PFS) **(A)** and overall survival (OS) **(B)** in stage IV lung cancer patients receiving ICIs. **(C, D)** Univariate analysis for identifying the prognosis factors significantly correlated with the PFS **(C)** and OS **(D)** in stage IV lung cancer patients receiving ICIs. **(E, F)** Multivariate analysis for identifying the significantly independent prognosis factors for PFS **(E)** and OS **(F)** in stage IV lung cancer patients receiving ICIs.

Supplementary Figure 5 Prognostic significance of C-PLAN index in immune checkpoint inhibitor (ICI) treated lung cancer patients with age over 65 years old. **(A, B)** Kaplan-Meier curves for the association of C-PLAN index with progression-free survival (PFS) **(A)** and overall survival (OS) **(B)** in ICI-treated lung cancer patients with age over 65 years old. **(C, D)** Univariate analysis for identifying the prognosis factors significantly correlated with the PFS **(C)** and OS **(D)** in ICI-treated lung cancer patients with age over 65 years old. **(E, F)** Multivariate analysis for identifying the significantly independent prognosis factors for PFS **(E)** and OS **(F)** in ICI-treated lung cancer patients with age over 65 years old.

Supplementary Figure 6 Prognostic significance of C-PLAN index in immune checkpoint inhibitor (ICI) treated lung cancer patients with age ranging from 18 to 65 years old. **(A, B)** Kaplan-Meier curves for the association of C-PLAN index with progression-free survival (PFS) **(A)** and overall survival (OS) **(B)** in ICI-treated lung cancer patients with age ranging from 18 to 65 years old. **(C, D)** Univariate analysis for identifying the prognosis factors significantly correlated with the PFS **(C)** and OS **(D)** in ICI-treated lung cancer patients with age ranging from 18 to 65 years old.
