# Supplementary figures and images for: Identification of C-PLAN index as a novel prognostic predictor for advanced lung cancer patients receiving immune checkpoint inhibitors
Source: Front Oncol. 2024 Feb 8;14:1339729. doi: 10.3389/fonc.2024.1339729 (PMC10883587; doi:10.3389/fonc.2024.1339729)

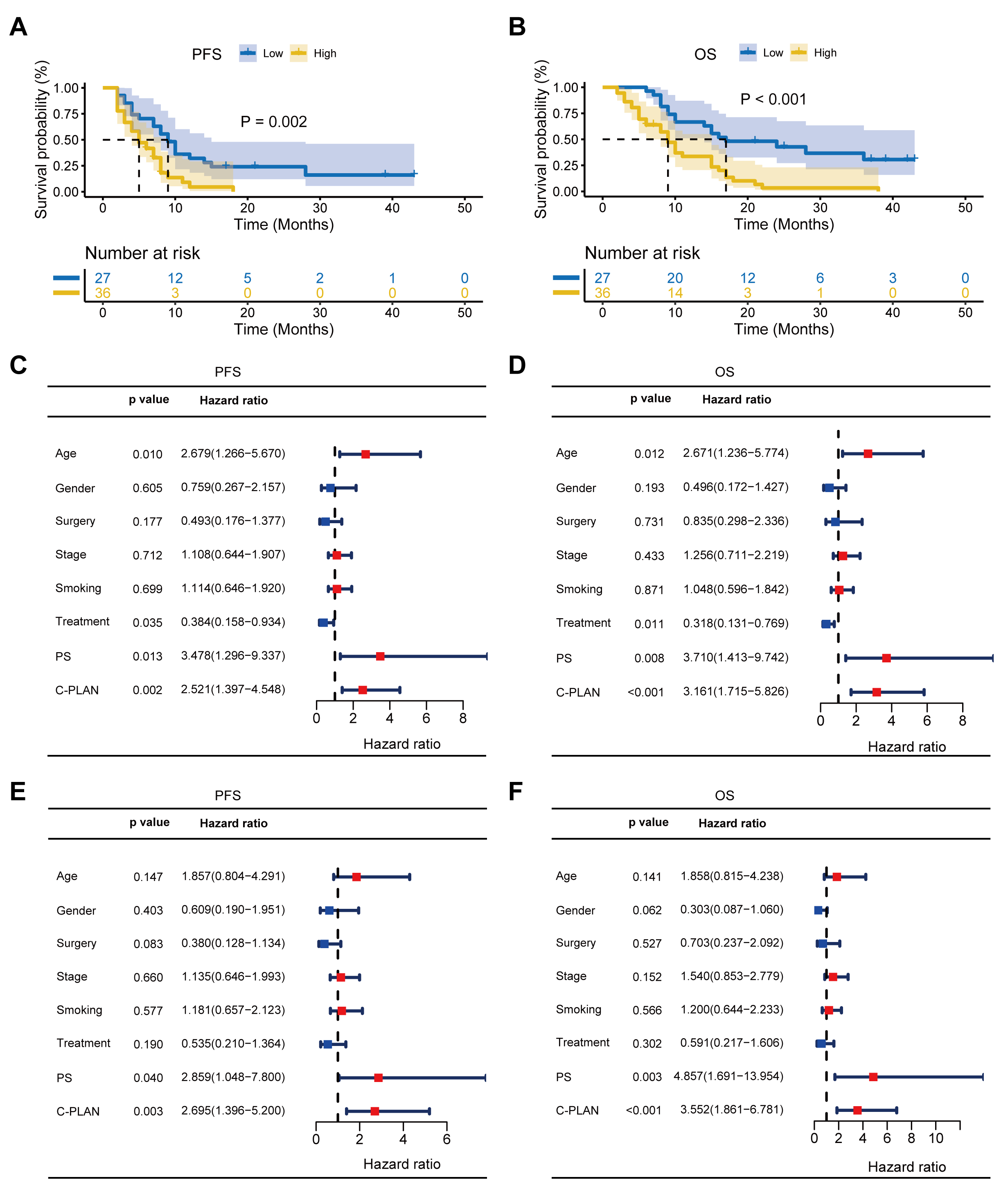

Supplement: Supplementary Figure 1 — Prognostic significance of C-PLAN index in small cell lung cancer (SCLC) patients receiving immune checkpoint inhibitors (ICIs). (A, B) Kaplan-Meier curves for the association of C-PLAN index with progression-free survival (PFS) (A) and overall survival (OS) (B) in SCLC patients receiving ICIs. (C, D) Univariate analysis for identifying the prognosis factors significantly correlated with the PFS (C) and OS (D) in SCLC patients receiving ICIs. (E, F) Multivariate analysis for identifying the significantly independent prognosis factors for PFS (E) and OS (F) in SCLC patients receiving ICIs. [file Image_1.tif]

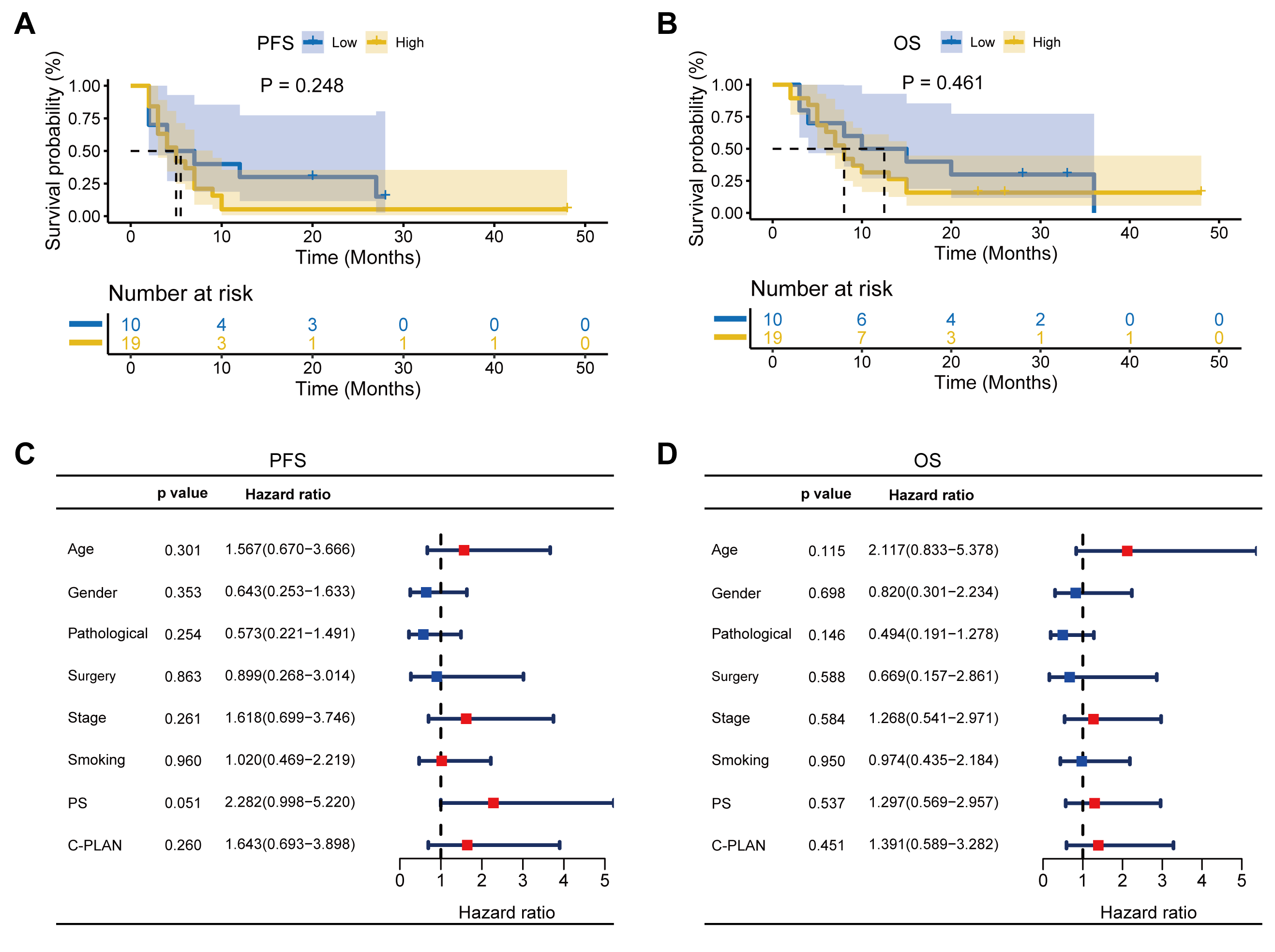

Supplement: Supplementary Figure 2 — Prognostic significance of C-PLAN index in advanced lung cancer patients receiving immune checkpoint inhibitor (ICI) monotherapy. (A, B) Kaplan-Meier curves for the association of C-PLAN index with progression-free survival (PFS) (A) and overall survival (OS) (B) in advanced lung cancer patients receiving ICI monotherapy. (C, D) Univariate analysis for identifying the prognosis factors significantly correlated with the PFS (C) and OS (D) in advanced lung cancer patients receiving ICI monotherapy. [file Image_2.tif]

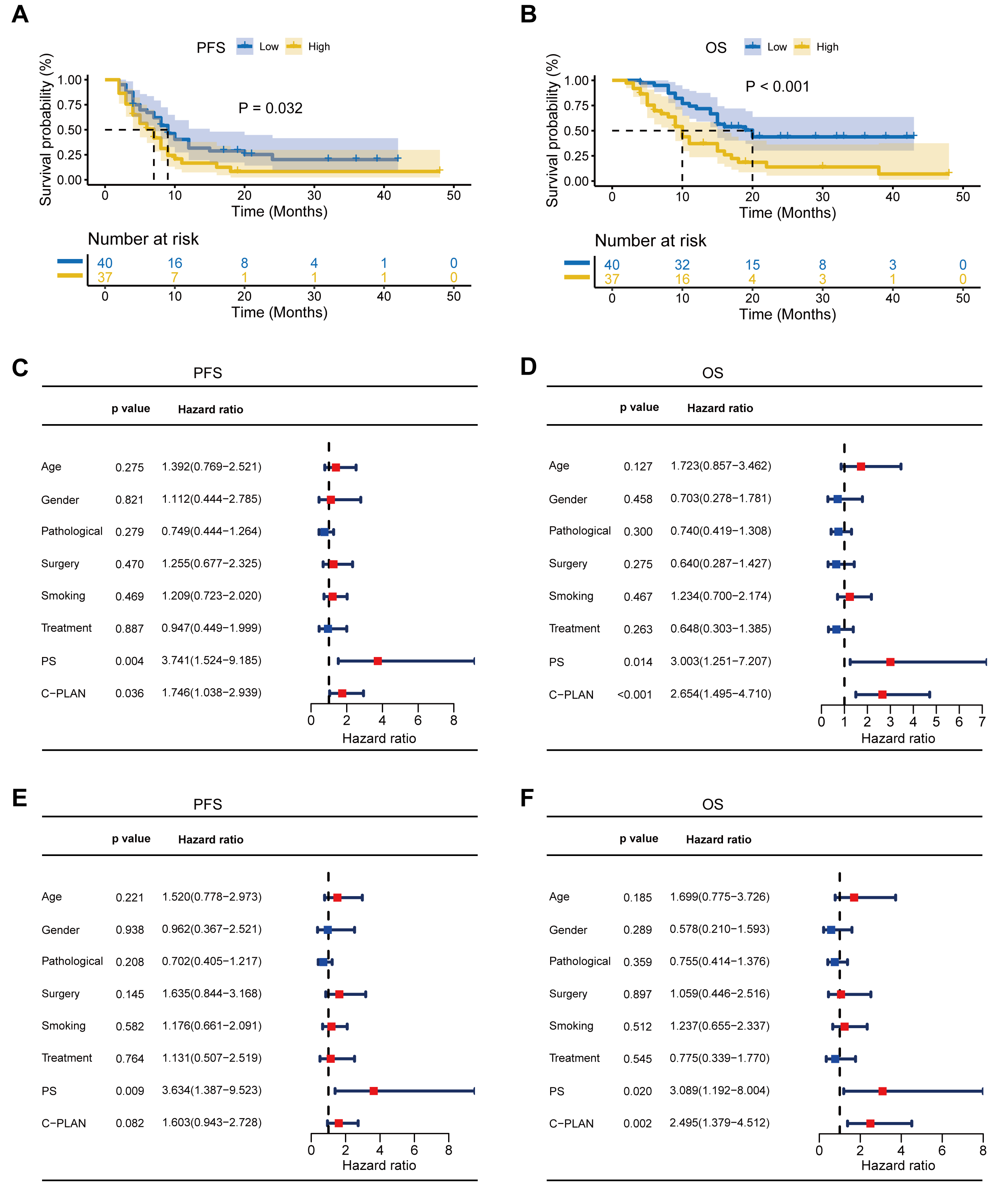

Supplement: Supplementary Figure 3 — Prognostic significance of C-PLAN index in stage III lung cancer patients receiving immune checkpoint inhibitors (ICIs). (A, B) Kaplan-Meier curves for the association of C-PLAN index with progression-free survival (PFS) (A) and overall survival (OS) (B) in stage III lung cancer patients receiving ICIs. (C, D) Univariate analysis for identifying the prognosis factors significantly correlated with the PFS (C) and OS (D) in stage III lung cancer patients receiving ICIs. (E, F) Multivariate analysis for identifying the significantly independent prognosis factors for PFS (E) and OS (F) in stage III lung cancer patients receiving ICIs. [file Image_3.tif]

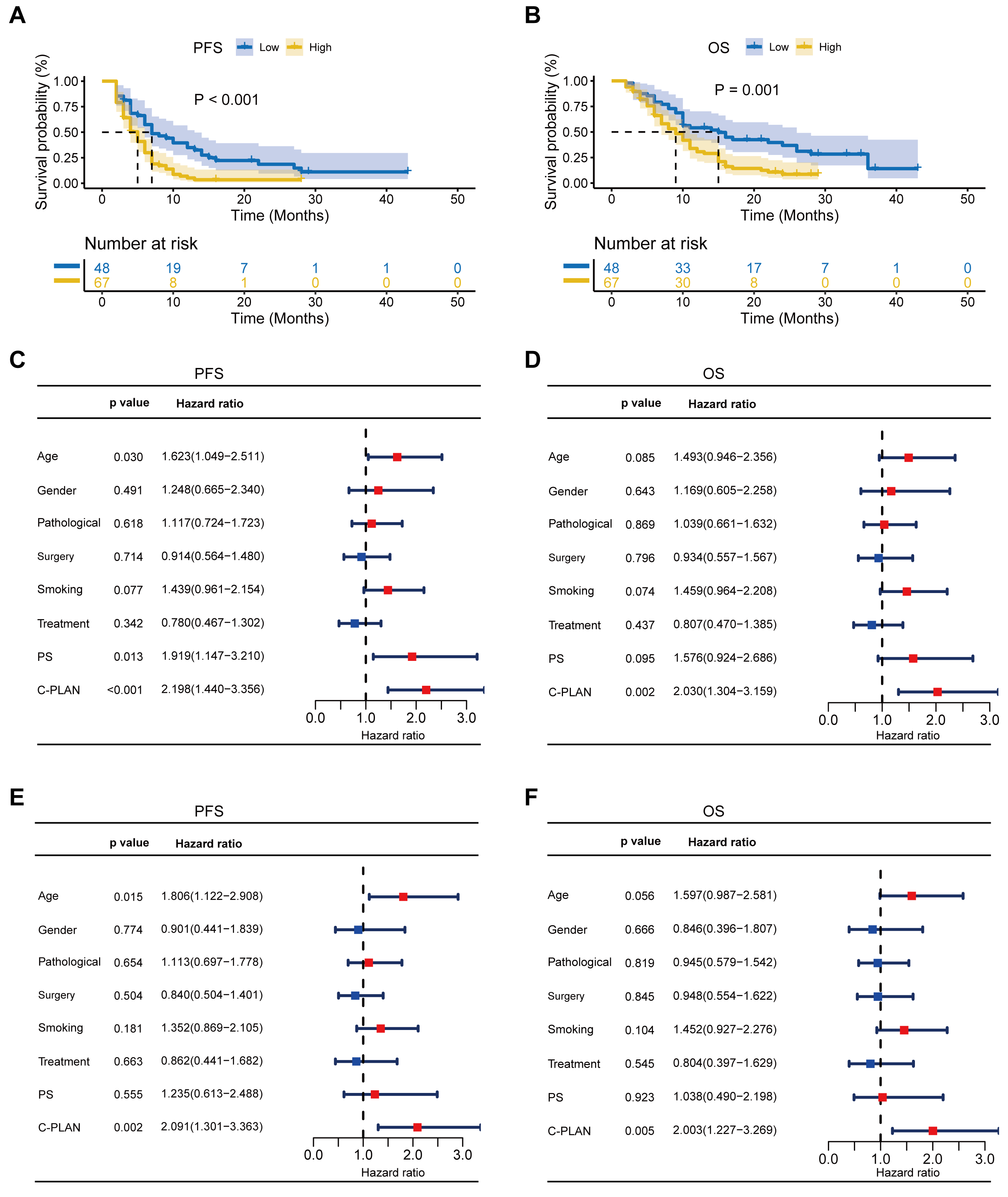

Supplement: Supplementary Figure 4 — Prognostic significance of C-PLAN index in stage IV lung cancer patients receiving immune checkpoint inhibitors (ICIs). (A, B) Kaplan-Meier curves for the association of C-PLAN index with progression-free survival (PFS) (A) and overall survival (OS) (B) in stage IV lung cancer patients receiving ICIs. (C, D) Univariate analysis for identifying the prognosis factors significantly correlated with the PFS (C) and OS (D) in stage IV lung cancer patients receiving ICIs. (E, F) Multivariate analysis for identifying the significantly independent prognosis factors for PFS (E) and OS (F) in stage IV lung cancer patients receiving ICIs. [file Image_4.tif]

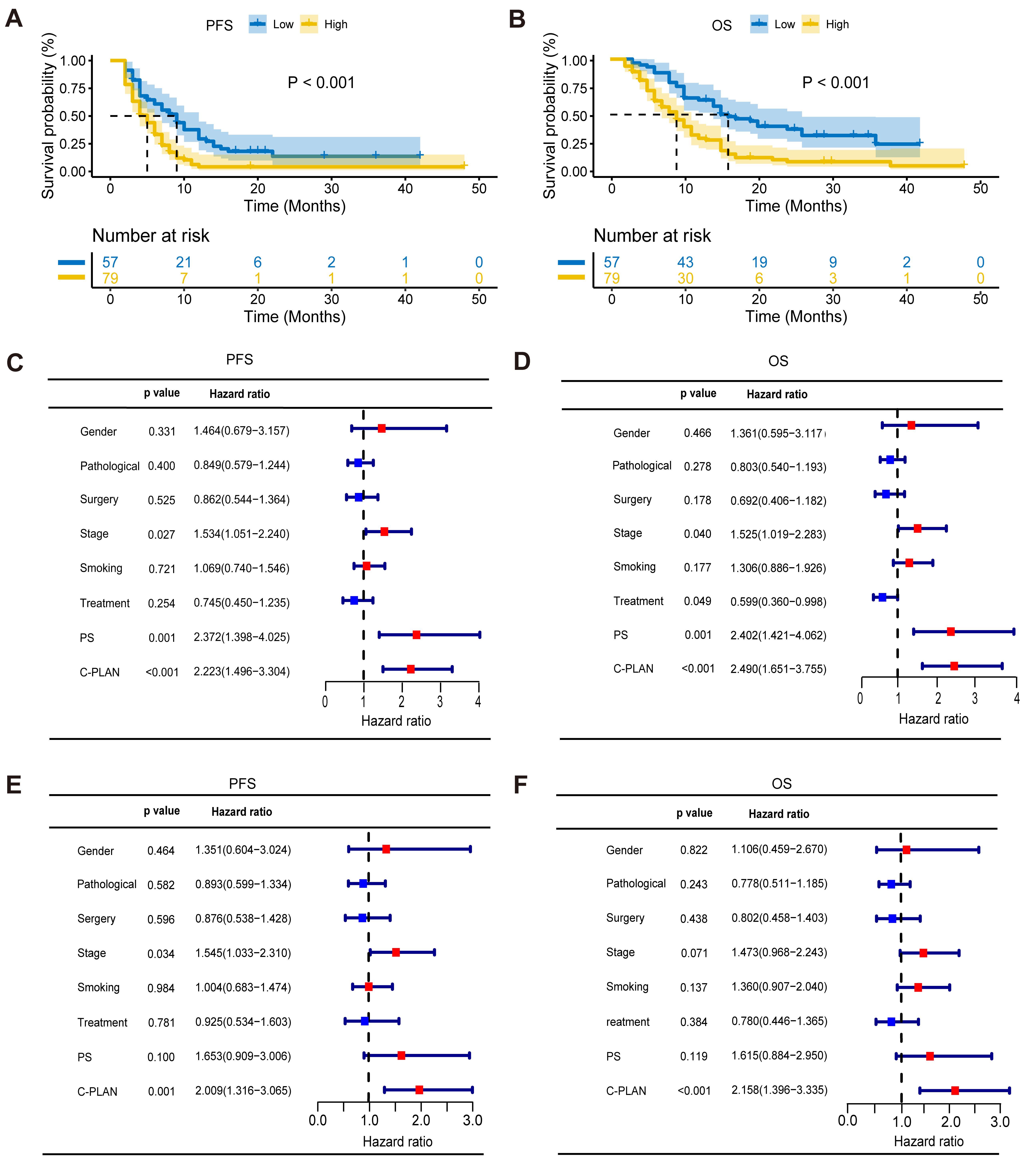

Supplement: Supplementary Figure 5 — Prognostic significance of C-PLAN index in immune checkpoint inhibitor (ICI) treated lung cancer patients with age over 65 years old. (A, B) Kaplan-Meier curves for the association of C-PLAN index with progression-free survival (PFS) (A) and overall survival (OS) (B) in ICI-treated lung cancer patients with age over 65 years old. (C, D) Univariate analysis for identifying the prognosis factors significantly correlated with the PFS (C) and OS (D) in ICI-treated lung cancer patients with age over 65 years old. (E, F) Multivariate analysis for identifying the significantly independent prognosis factors for PFS (E) and OS (F) in ICI-treated lung cancer patients with age over 65 years old. [file Image_5.tif]

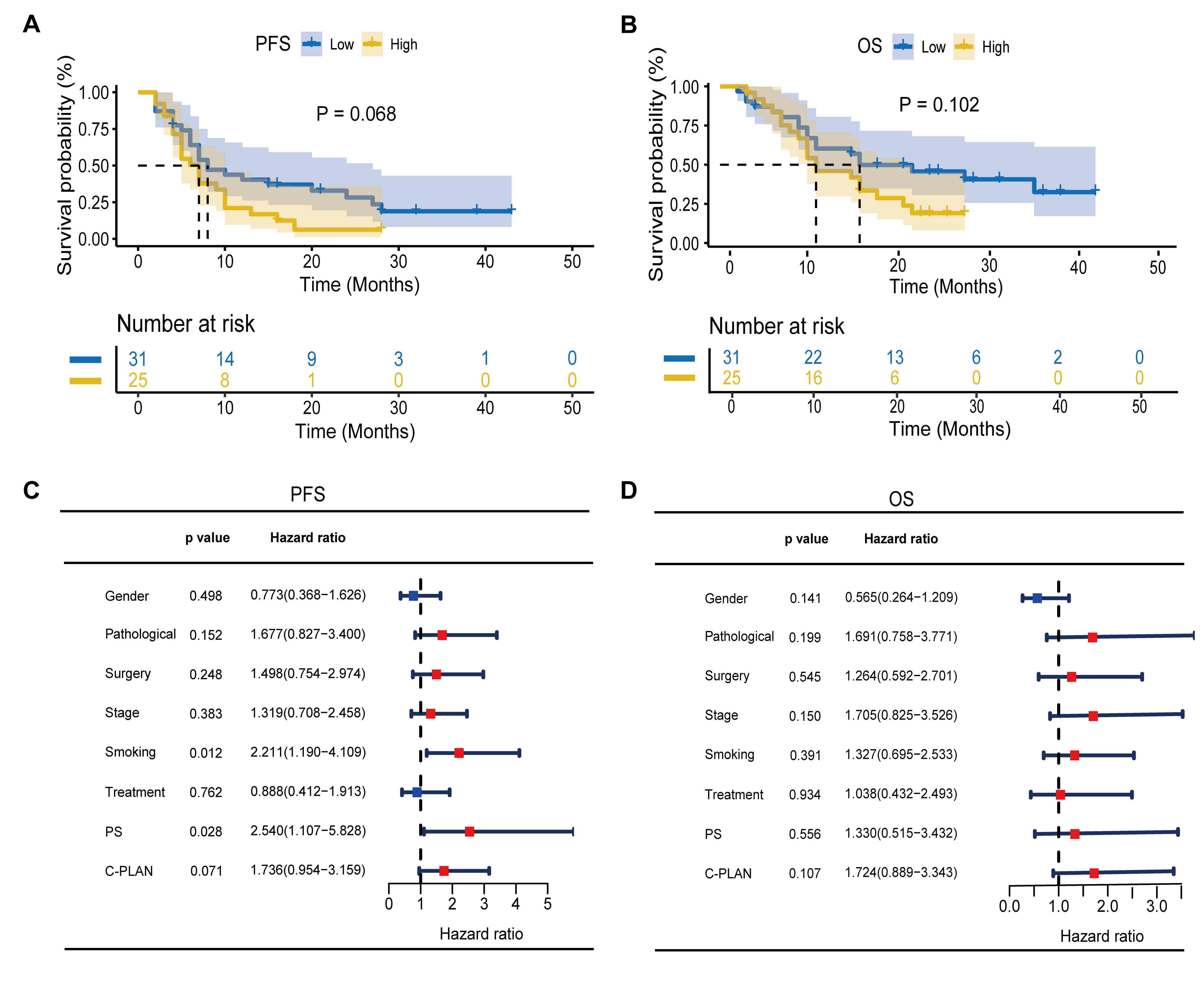

Supplement: Supplementary Figure 6 — Prognostic significance of C-PLAN index in immune checkpoint inhibitor (ICI) treated lung cancer patients with age ranging from 18 to 65 years old. (A, B) Kaplan-Meier curves for the association of C-PLAN index with progression-free survival (PFS) (A) and overall survival (OS) (B) in ICI-treated lung cancer patients with age ranging from 18 to 65 years old. (C, D) Univariate analysis for identifying the prognosis factors significantly correlated with the PFS (C) and OS (D) in ICI-treated lung cancer patients with age ranging from 18 to 65 years old. [file Image_6.tif]
